# Mesh Rectopexy or Resection Rectopexy for Rectal Prolapse; Is There a Gold Standard Method: A Systematic Review, Meta-Analysis and Trial Sequential Analysis

**DOI:** 10.3390/jcm13051363

**Published:** 2024-02-28

**Authors:** Georgios Koimtzis, Leandros Stefanopoulos, Georgios Geropoulos, Christopher G. Chalklin, Ioannis Karniadakis, Awad A. Alawad, Vyron Alexandrou, Nikos Tteralli, Eliot Carrington-Windo, Andreas Papacharalampous, Kyriakos Psarras

**Affiliations:** 1 Department of Oesophageal and Gastric Surgery, University Hospital of Wales, Cardiff and Vale University Health Board, Cardiff CF14 4XW, UK; 2 Department of Electrical and Computer Engineering, Northwestern University, 633 Clark St., Evanston, IL 60208, USA; leandros@northwestern.edu; 3 Western General Hospital, NHS Lothian, Crewe Road South, Edinburgh EH4 2XU, UK; georgios.geropoulos@nhs.net; 4 Cardiff Transplant Unit, University Hospital of Wales, Cardiff and Vale University Health Board, Cardiff CF14 4XW, UK; christopher.chalklin@wales.nhs.uk (C.G.C.); ioannis.karniadakis@wales.nhs.uk (I.K.); awad.alawad@wales.nhs.uk (A.A.A.); 5 Urology Department, General Hospital of Thessaloniki “G. Gennimata-Agios Dimitrios”, Elenis Zografou 2, 54634 Thessaloniki, Greece; vyrwnal@hotmail.com; 6 Department of General Surgery, North Hampshire NHS Foundation Trust, Basingstoke RG24 9NA, UK; nikos.tteralli@hhft.nhs.uk; 7 Department of General Surgery, Grange University Hospital, Caerleon Road, Llanfrechfa, Cwmbran NP44 8YN, UK; eliot.carrington-windo@wales.nhs.uk; 8 Department of Surgery Larnaca General Hospital Pandoras, Larnaca 6301, Cyprus; andreaspch96@hotmail.com; 9 School of Medicine, Second Surgical Propedeutic Department, Ippokrateio General Hospital, Aristotle University of Thessaloniki, Konstantinoupoleos 49, 54642 Thessaloniki, Greece; psarrask@auth.gr

**Keywords:** rectal prolapse, resection rectopexy, mesh rectopexy

## Abstract

(1) Background: Rectal prolapse is a benign condition that mainly affects females and the elderly. The most common symptoms are constipation and incontinence. The treatment of choice is surgical, but so far, there has been no gold standard method. The aim of this study is to compare the two most common intrabdominal procedures utilized for treating rectal prolapse: the resection rectopexy and the mesh rectopexy. (2) Methods: In this study, we conducted a thorough systematic review and meta-analysis of the available literature and compared the two different approaches regarding their complication rate, recurrence rate, and improvement of symptoms rate. (3) Results: No statistically significant difference between the two methods was found regarding the operating time, the length of stay, the overall complication rate, the surgical site infection rate, the cardiopulmonary complication rate, the improvement in constipation and incontinence rates, and the recurrence rate. (4) Conclusions: Our study revealed that mesh rectopexy and resection rectopexy for rectal prolapse have similar short- and long-term outcomes. As a result, the decision for the procedure used should be individualized and based on the surgeon’s preference and expertise.

## 1. Introduction

Rectal prolapse is a rare, benign disease that has an incidence of 0.5% in the general population. It mainly affects females and the elderly. It is defined as the full-thickness protrusion of the rectal wall through the anus (external prolapse) [1]. On the other hand, internal rectal prolapse is defined as intussusception of the rectum above the level of the sphincteric mechanism [1]. The most predominant risk factors for rectal prolapse include old age, straining, traumatic vaginal delivery, or multiple vaginal deliveries. Nonetheless, in younger patients, the risk factors include chronic psychiatric diseases, previous pelvic surgery, redundant sigmoid colon, inflammatory bowel disease or colitis, irritable bowel syndrome, family history of gastrointestinal diseases, uterovaginal prolapse, solitary rectal ulcer, and Ehlers–Danlos syndrome [2]. Usually, rectal prolapse presents with fecal incontinence, constipation, or both, while it is also associated with blood and mucous discharge from the anus [3]. The diagnosis and assessment of external rectal prolapse do not usually require any specific diagnostic investigation, apart from the cases where fecal incontinence is present. Nonetheless, in the assessment of internal rectal prolapse, various diagnostic modalities may be useful, such as barium dynamic assessment and magnetic resonance or isotope defecography. Treatment of rectal prolapse is mainly surgical. Currently, there have been more than 120 techniques described in the literature, but there is a lack of consensus regarding the best available option, and no gold standard method has been suggested so far [4,5]. However, only a few procedures are routinely applied [1]. Generally, these procedures can be separated into two large categories: the ones that are carried out using a peritoneal approach and the ones that are performed through a perineal approach. The latest guidelines published by the American Society of Colorectal Surgeons suggest that for patients with satisfactory performance status and acceptable risk, a peritoneal approach should be used as it leads to lower recurrence rates and more satisfactory functional outcomes [1]. Nowadays, the most frequently used peritoneal procedures include the resection rectopexy and mesh rectopexy [4,5,6,7,8]. 

These operations can be carried out by open, laparoscopic, or robotic approach [9,10]. All of them include elevation of the rectum out of the pelvis in order to correct the protrusion, followed by stabilization (rectopexy) with different methods on the presacral fascia [2]. Resection rectopexy, also known as the Frykman–Goldberg procedure [11], consists of sigmoidectomy, tension-free anastomosis of the colon, and rectopexy with sutures on the presacral fascia [10]. During this procedure, the mesorectum is initially dissected to the level of the pelvic floor, both anteriorly and posteriorly. The level reached corresponds to the upper edge of the external sphincter, while the lateral ligaments are left completely intact. After the resection is complete, the Douglas’ pouch or the rectovesical space is reconstructed by suturing the peritoneum to the right and left of the rectum. The rectopexy is completed by suturing the anterior rectal wall to the peritoneum. Mesh rectopexy without resection is another alternative that consists of a mesh or biological graft placement to reinforce either the anterior rectum or to fixate the rectum on the sacrum [12,13,14,15]. In this procedure, the initial dissection is similar to the one carried out for the resection rectopexy. However, after the level of the pelvic floor muscles is reached, a mesh is used to complete the rectopexy. The mesh is usually fashioned in a spatula shape and is placed anteriorly to the rectum. It is then fixated to the sides of the rectum using absorbable sutures and then fixated to the sacral promontory. The adhesive material can also be used to secure the mesh to the anterior rectal wall. Finally, the mesh is covered by approximating the pelvic peritoneum.

The purpose of our current study is to assess whether one of the above-mentioned procedures (resection rectopexy or mesh rectopexy) has better outcomes than the other. These procedures were compared regarding their complication rate, recurrence rate, and the patients’ quality of life postoperatively (constipation and fecal incontinence improvement).

## 2. Materials and Methods

This systematic review and meta-analysis were carried out without a pre-existing registered protocol. It has been prepared by strictly adhering to the PRISMA checklist. A thorough and systematic literature search was performed to identify studies that compared the postoperative outcomes of resection rectopexy and mesh rectopexy for treating rectal prolapse. The databases that were looked into for relevant studies published in English were MEDLINE, Scopus, and Cochrane Library databases until 31 May 2023. An additional search to identify any available grey literature was carried out on the websites of international colorectal associations and on the abstract books of relevant conferences. The search of the MEDLINE database was carried out using the following search string: ((rectal prolapse[MeSH Terms]) AND (resection rectopexy)) AND (mesh rectopexy). Similar search strings were used for the other databases. 

Firstly, two independent researchers (I.K. and G.G.) performed a detailed search of the above-mentioned databases. The inclusion criteria to which the generated studies were compared to were the following: (1) studies performed on human patients; (2) patients suffering from rectal prolapse undergoing operative treatment with either resection or mesh rectopexy; (3) articles written in English; and (4) articles having sufficient and extractable data on the operating time, length of stay, complication rate, recurrence rate and constipation and incontinence improvement rate. When there was a case of disagreement between the two reviewers, another experienced reviewer (N.T.) provided their opinion, and the ultimate decision on these studies was based on either a consensus or the majority opinion.

Data extraction was performed by two independent members of the research team (V.A. and E.C.-W.), and their findings were confirmed by a third assessor (A.P.). Extracted data from each article include the first author’s name, the publication date, the study design, the number of patients included, the patients’ demographics (age and sex), the type of procedure they underwent, the operating time, the length of stay, the overall complication rate, the surgical site infection rate, the cardiopulmonary complication rate, the recurrence rate, the improvement of constipation and improvement of incontinence rate and the mortality rate.

In this meta-analysis, all statistical analyses were cοnducted by utilizing Reviewer Manager 5.4.1 software [Review Manager (RevMan) (computer program) version 5.4.1, Copenhagen: The Nordic Cochrane Centre, Denmark, the Cochrane Collaboration, 2020] and STATA version 16.1. The data in this study are presented as mean ± standard deviation, while odds ratios (ORs) and weighted mean differences (WMDs) with a confidence interval (CI) of 95% were calculated for dichotomous and continuous variables, respectively. The level of statistical significance was set at a *p*-value of less than 0.05. When large heterogeneity among the studies (I^2^ ≥ 50%) was present, a random effects model was applied, while in cases of low heterogeneity, a fixed effects model was utilized. When a random effects model was applied, sensitivity analysis was carried out at the levels of I^2^ = 50% and I^2^ = 25% to assess the effect of the large heterogeneity of the studies on the outcome of the meta-analysis. The publication bias was assessed by designing the respective Begg’s funnel plot. Trial sequential analysis (TSA) was also carried out to evaluate whether the sample size in each analysis was enough to yield valid results or if further studies were needed. The software used to conduct TSA was Trial Sequential Analysis (TSA) (computer program) version 0.9.5.10 beta, Copenhagen Trial Unit, Centre for Clinical Intervention Research, Capital Region, Copenhagen University Hospital—Rigshospitalet, 2021.

The potential risk of bias across the studies that were included in this systematic review and meta-analysis was assessed using the ‘Cochrane Collaboration tool for assessing the risk of bias’ as integrated into the Review Manager 5.4.1 software, and the outcomes are presented in a risk of bias graph and a risk of bias summary.

## 3. Results

**Figure 1:**
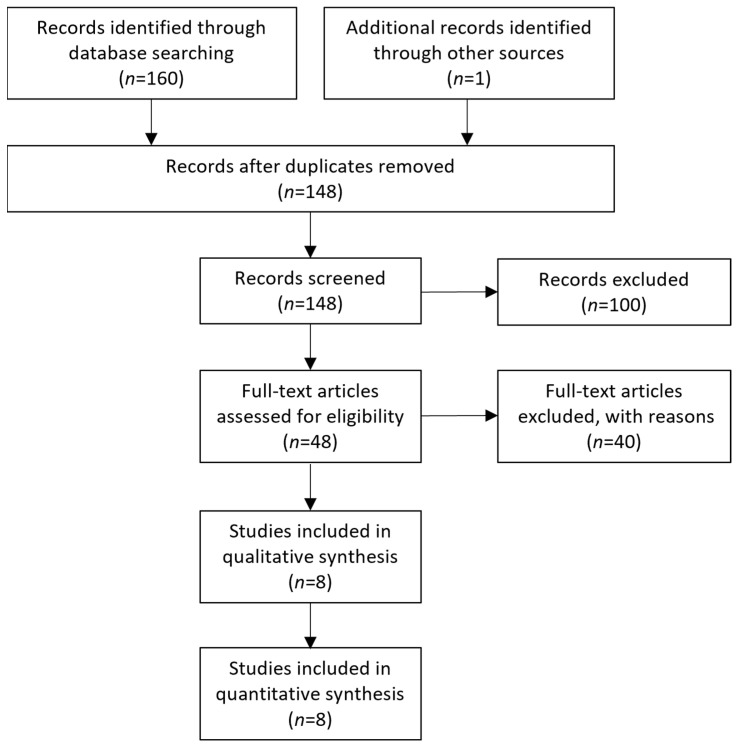
PRISMA flowchart depicting the study selection method.

The initial literature search of the online archives resulted in a collective number of 160 articles, while one more was revealed through the search for grey literature. Following the removal of duplicates, the number of articles was brought down to 148. Afterward, the identified studies were screened according to their title and abstract, ultimately resulting in 48 articles eligible for full-text analysis. The remaining articles were not included because they investigated different research questions to the one of our study, or on the grounds of including non-adult patients, or because the required data were not extractable. Following the full-text analysis, eight studies in total [12,13,14,15,16,17,18,19] were eligible for inclusion in the qualitative and quantitative analysis. The selection process of the included articles can be found in Figure 1.

The manuscripts included in the final analysis were published between 1992 and 2018. Three of these studies were prospective and randomized, while the rest were retrospective. The total number of patients included in these studies was 483, with 207 undergoing resection rectopexy and 276 undergoing mesh rectopexy. The basic characteristics of each individual article can be found in Table 1.

**Figure 2:**
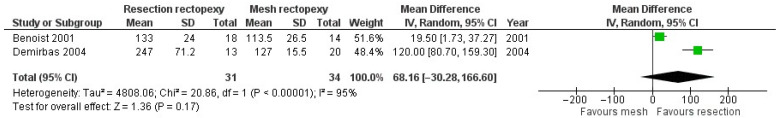
Forest plot of the analysis of the operative time of the compared procedures [13,14].

**Figure 3:**
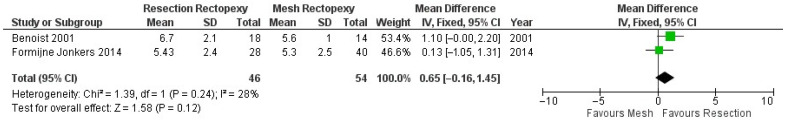
Forest plot of the analysis of the length of stay of the compared procedures [13,18].

Two [11,12] out of the eight selected studies had extractable data on the operating time of the procedures carried out, and our statistical analysis showed that the two methods did not differ significantly (WMD 68.16, 95% CI −30.28 to 166.6). This analysis is shown in Figure 2. However, the sensitivity analysis of this random effect model with I^2^ = 50% and I^2^ = 25%, revealed a statistically significant difference indicating longer operative time in cases of resection (WMD 53.15, 95% CI 24.37 to 81.93, *p* < 0.001 and WMD 44.85, 95% CI 23.22 to 66.48, *p* < 0.001, respectively). Similarly, two studies [11,16] provided data on the length of stay, and the meta-analysis revealed similar outcomes between the two methods (WMD 0.65, 95% CI −0.16 to 1.45). This outcome is shown in Figure 3. 

**Figure 4:**
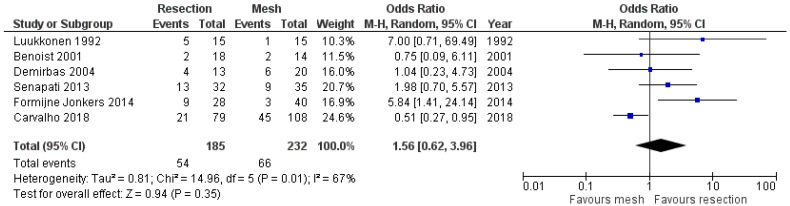
Forest plot demonstrating the comparison of the overall complication rate [12,13,14,15,18,19].

**Figure 5:**
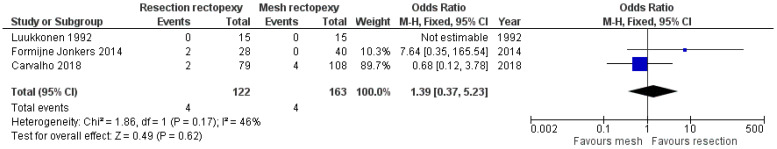
Forest plot demonstrating the comparison of the surgical site infection rate [12,15,18].

**Figure 6:**
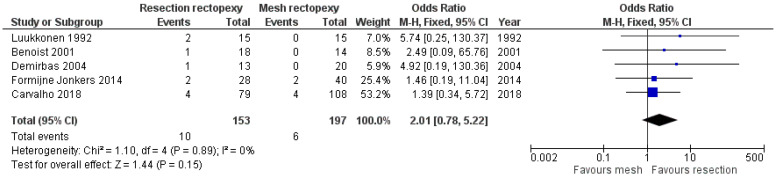
Forest plot demonstrating the comparison of the cardiopulmonary complication rate [12,13,14,15,18].

Regarding the overall complication rate, six studies provided data [10,11,12,13,16,17], and the meta-analysis showed that the two methods did not differ significantly (OR 1.56, 95% CI 0.62 to 3.96). This finding is demonstrated in Figure 4. The sensitivity analysis of this random effects model meta-analysis with I^2^ = 50% and I^2^ = 25% confirmed the above findings (logOR −0.37, 95% CI −1.13 to 0.38, *p* = 0.334 and logOR −0.23, CI 95% −0.83 to 0.35, *p* = 0.433, respectively). Also, a statistical analysis of the rate of surgical site infections that was mentioned in three studies showed that the two methods under study had similar outcomes (OR 1.39, 95% CI 0.37 to 5.23). This outcome is shown In Figure 5. Furthermore, the comparison of the cardiopulmonary complication rate that was mentioned in five studies showed that the two methods under investigation did not differ significantly (OR 2.01, 95% CI 0.78 to 5.22). This outcome is shown in Figure 6. Moreover, only one case of death was reported by Luukkonen et al. in the resection group on the second postoperative day due to myocardial infarction.

**Figure 7:**
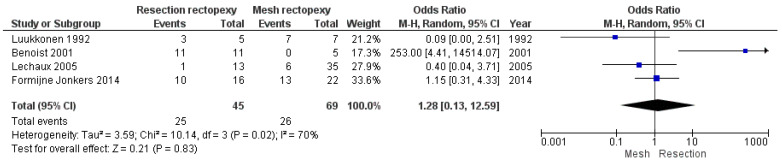
Forest plot of the analysis of the constipation improvement rate [13,15,17,18].

**Figure 8:**
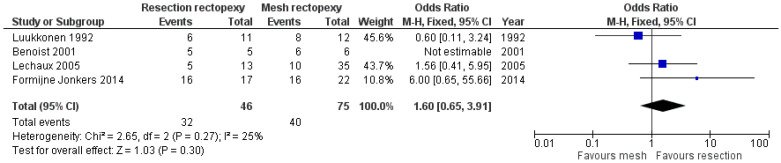
Forest plot of the comparison of the incontinence improvement rate [13,15,17,18].

**Figure 9:**
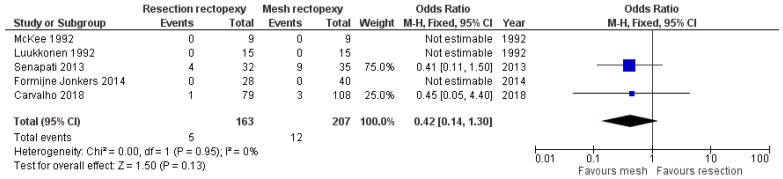
Forest plot of the comparison of the recurrence rate [12,15,16,18,19].

Regarding the long-term post-operative outcomes, there are comparable outcomes in the rate of constipation improvement between the two methods (OR 12.59, 95% CI 0.13 to 12.59). This outcome is shown in Figure 7. As this meta-analysis was carried out with an random effects model due to the large heterogeneity, we conducted a further sensitivity analysis with I^2^ = 50% and I^2^ = 25%, which confirmed our findings (logOR −0.123, CI 95% −0.186 to 1.61 *p* = 0.889 and logOR −0.04, CI 95% −1.38 to 1.30, *p* = 0.953). Moreover, the two techniques had comparable outcomes in the rate of incontinence improvement (OR 1.60, 95% CI 0.65 to 3.91). This outcome is shown in Figure 8. Finally, no statistically significant difference between the two methods was identified when comparing the recurrence rate (OR 0.42, 95% CI 0.14 to 1.30). This comparison is demonstrated in Figure 9.

**Figure 10:**
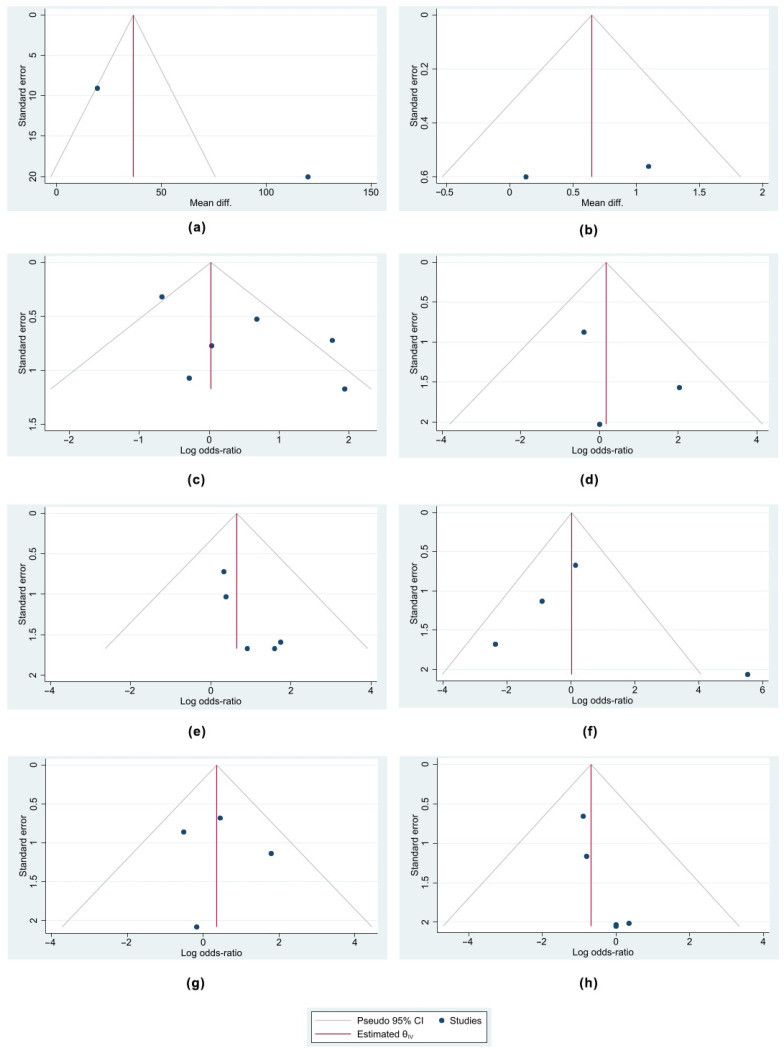
Funnel plots of the analyses performed indicating that there was no publication bias among the selected studies. (**a**) Funnel plot of comparison of operative time, (**b**) funnel plot of comparison of length of stay, (**c**) funnel plot of comparison of overall complication rate, (**d**) funnel plot of comparison of surgical site infection rate, (**e**) funnel plot of comparison of cardiopulmonary complication rate, (**f**) funnel plot of comparison of constipation improvement rate, (**g**) funnel plot of comparison of incontinence improvement rate, and (**h**) funnel plot of comparison of recurrence rate.

**Figure 11:**
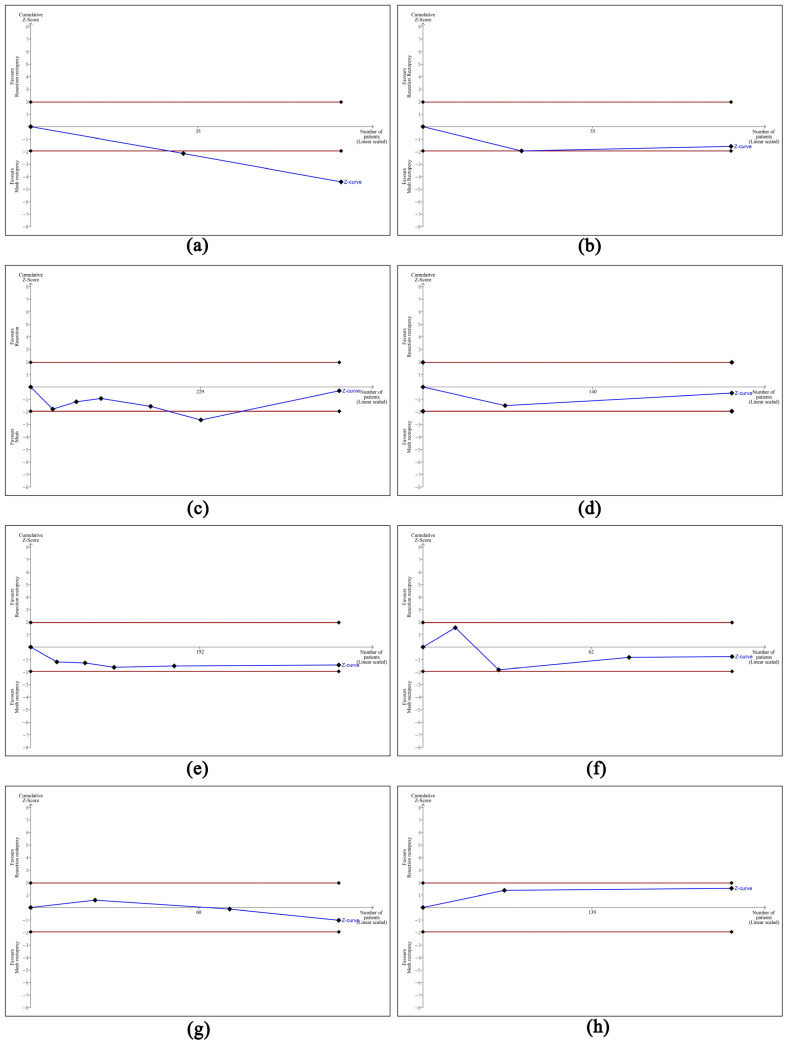
Outcomes of trial sequential analysis showing that the number of the included studies was sufficient to draw definite conclusions only for the first meta-analysis. (**a**) TSA of meta-analysis of operative time, (**b**) TSA of meta-analysis of length of stay, (**c**) TSA of meta-analysis of overall complication rate, (**d**) TSA of meta-analysis of surgical site infection rate, (**e**) TSA of meta-analysis of cardiopulmonary complication rate, (**f**) TSA of meta-analysis of constipation improvement rate, (**g**) TSA of meta-analysis of incontinence improvement rate, and (**h**) TSA of meta-analysis of recurrence rate.

Based on the designed Funnel plots, no publication bias was identified across all the studies selected in all eight analyses performed. These outcomes are portrayed in Figure 10. Nonetheless, the trial sequential analysis performed revealed that more studies are required to corroborate our findings for all the comparisons made, apart from the operative time where the number of patients was sufficient to draw valid conclusions. The outcomes of the trial sequential analysis are shown in Figure 11.

**Figure 12:**
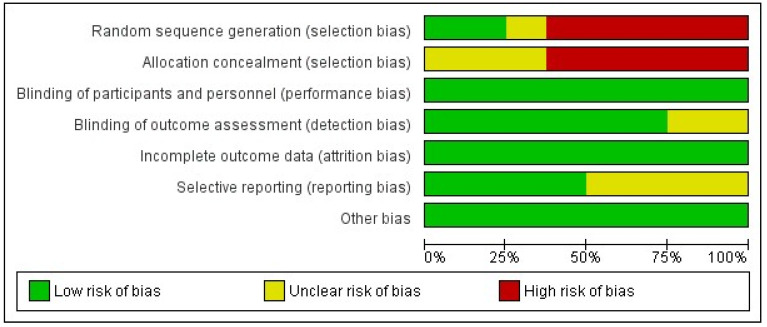
Risk of bias graph showing the percentage of bias that each study introduced in our meta-analysis.

**Figure 13:**
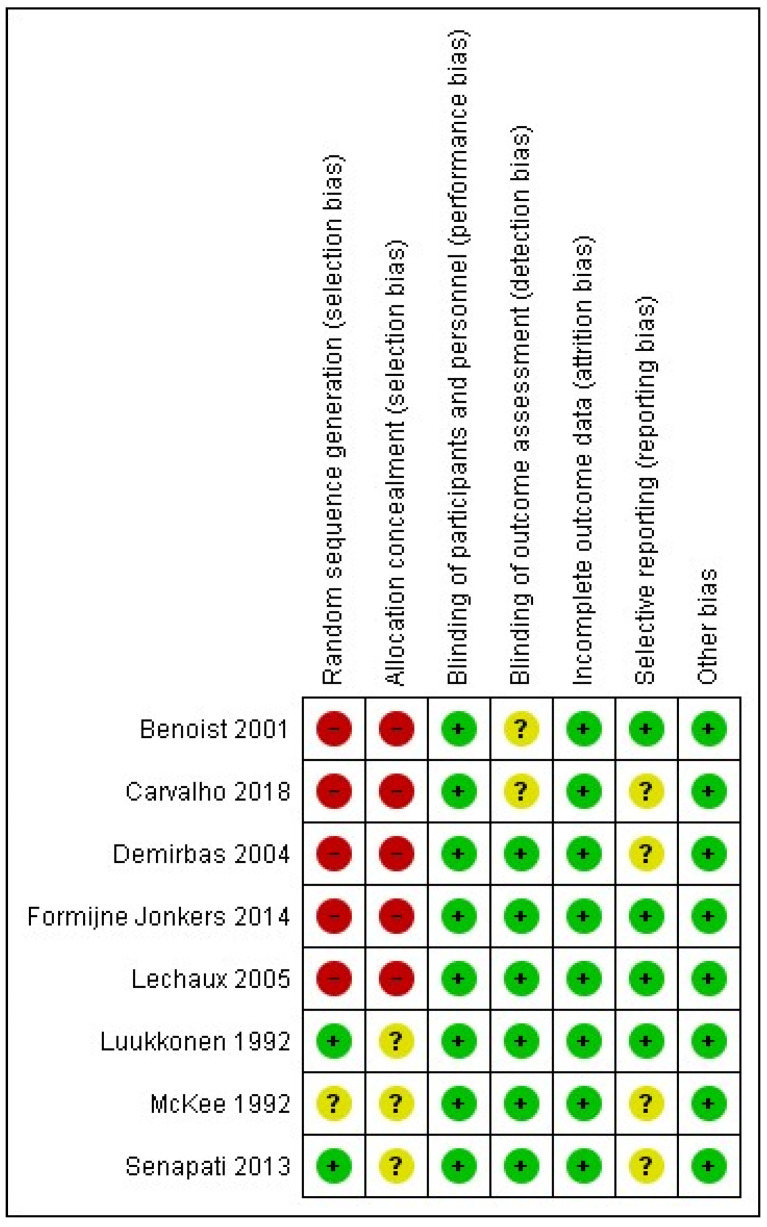
Risk of bias summary showing the level of risk of bias for each study included in the meta-analysis [12,13,14,15,16,17,18,19].

Finally, the outcome of the assessment of potential bias in the included studies in this systematic review and meta-analysis is shown in Figure 12 and Figure 13. According to this assessment, there is a significant risk of selection bias due to inadequate randomization and inadequate concealment prior to the intervention. This could have potentially affected the outcomes as the interventions were either based on availability, the surgeon’s preference, or on the patient’s specific symptoms as part of an individualized approach. Nonetheless, despite the lack of blindness, there is a low risk of performance and detection bias as the outcomes were judged using various valid scoring systems of post-operative performance or they were assessed using investigations such as anal manometry. Finally, there is a low risk of attrition, and as there were no missing outcome data, the risk of reporting bias is low to unclear as half of the included studies did not report all of the outcomes that were of interest in our systematic review and meta-analysis. 

## 4. Discussion

Based on our review of the literature and as far as we can tell, this is the first meta-analysis that specifically compares the outcomes of resection rectopexy to mesh rectopexy for the management of rectal prolapse. Our results show that both procedures have similar outcomes regarding operative time, length of stay, recurrence rate, and complication rate, as well as similar outcomes regarding improvement of fecal incontinence and constipation. Nonetheless, the trial sequential analysis demonstrated that more studies will be required to confirm our findings. As a result, each patient will require an individualized approach, and the decision will ultimately rest with the operative surgeon and their personal experience and preference regarding the procedure of choice. 

According to our review of the literature, so far, there have been some systematic reviews for the operative management of complete rectal prolapse that include abdominal approaches, but most of them compare other techniques, such as ventral mesh rectopexy and suture rectopexy (without sigmoidectomy), or they compare different approaches (mainly robotic and laparoscopic). From those that include abdominal approaches, Tou S. et al. [3] conclude that there are still not enough data to conclude which of the abdominal procedures is more effective. Although they conclude that bowel resection was associated with lower constipation rates, they believe that the usefulness of their results to guide clinical decisions is limited due to some limitations of the included studies. Another systematic review by Hotouras et al. in 2015 on the operative management of recurrent rectal prolapse was unable to come up with a management algorithm for recurrent rectal prolapse as a result of the wide variety of surgical procedures utilized and the low level of evidence within heterogeneous articles [20]. However, they report recurrence rates from 0% to 15% for abdominal procedures, with morbidity rates ranging from 0% to 32% and a mortality of 4%. Another systematic review by Faucheron et al. [21] included twelve case series with a total number of 574 patients that underwent laparoscopic anterior rectopexy and reported a mean recurrence rate of 4.7% with a median follow-up of 23 months. Also, constipation improved within a range of 3–72%, but deterioration or new appearance of constipation occurred in 0–20%. Incontinence improved in 31–84% of the patients. Moreover, in another meta-analysis of eight studies with a total number of 467 patients performed by Cadeddu et al., there were similar outcomes in recurrence, incontinence, and constipation improvement rates between laparoscopic and open abdominal rectopexy [22]. Also, regarding non-comparative trials, there was no statistically significant difference in recurrence rate in open and laparoscopic suture rectopexy studies and in open and laparoscopic mesh rectopexy trials [20]. Furthermore, another meta-analysis of 5 comparative studies by Hajibandeh et al. showed that laparoscopic mesh rectopexy has a lower recurrence rate but longer procedure time when compared to laparoscopic posterior suture rectopexy [23]. Finally, regarding the optimal method of approach, laparoscopic surgery has comparable outcomes with open in terms of morbidity and recurrence rate, while it has a shorter hospital stay [24]. The robotic approach has been revealed to have equal post-operative outcomes and an even shorter hospital stay [25,26]. 

Another interesting study that focused on the management of rectal prolapse in men was carried out by Poylin et al. in 2019 [27]. According to this multicenter retrospective review of 58 male patients who underwent surgical repair for rectal prolapse, thirty-nine (67%) patients underwent an abdominal procedure. These patients were younger and had a lower American Society of Anesthesiologists (ASA) score. The overall complication rate in this study was 26%, with the most common complication being urinary retention (16%). However, this was more common in perineal procedures. Also, the overall recurrence rate was 9%, with similar outcomes between the abdominal and perineal procedures. Regarding the long-term outcomes of this study, the constipation rate decreased from 59% to 36%, and fecal incontinence decreased from 40% to 14%. Nonetheless, 5% of the patients reported a new onset of constipation, and 7% developed new symptoms of incontinence. On top of that, 3% of the patients reported post-operative symptoms of sexual dysfunction. This study concludes that although surgical repair of rectal prolapse in men is a safe surgical procedure with a low recurrence rate, more studies are needed to identify which is the best surgical approach [26]. Another study that focused on the management of rectal prolapse in male patients was published in 2022 by Hu et al. [28]. This study performed a retrospective comparison between abdominal and perineal procedures for the management of external rectal prolapse. It included a total number of 51 patients and ultimately revealed that a perineal approach, either Altemeier or Delorme procedure, carries a higher complication and recurrence rate. Also, regarding the long-term functional outcomes, constipation was improved in both approaches, but fecal incontinence deteriorated with an abdominal approach. Nonetheless, patients in both groups reported an overall improvement in their quality of life, as assessed by the EuroQol 5-Dimension 5-Levels quality of life questionnaire. The matter of the management of rectal prolapse in male patients was also investigated by Ganapathi et al., who also published the outcomes of their study in 2022 [29]. They compared the outcomes of modified laparoscopic posterior mesh rectopexy (LPMR) to the ones of laparoscopic resection rectopexy (LRR) on a total number of 118 male patients. According to their findings, the mean operative time for LPMR was 102 ± 22 min and 121 ± 26 min for LRR, while the length of stay was 4.6 ± 1.4 days and 6.3 ± 1.2 days, respectively. Also, there were 12 cases of complications in the LPMR group and 5 cases of complications in the LRR group. Finally, two patients in each group reported post-operative constipation that improved with laxatives. The authors of this study conclude that randomized trials comparing these two methods will be required to establish if one of them is superior to the other.

Recently, an alternative to the classic operative approach for the management of rectal mucosal prolapse has been reported by Liu et al. [30]. The authors have reported the use of cap-assisted endoscopy sclerotherapy (CAES) to treat outlet obstructive constipation caused either by internal hemorrhoids or rectal mucosal prolapse. In this technique, a sclerosing agent is injected above the dental line at the area of loose submucosa via a colonoscope bearing a regular cap at its top. Based on the results of the pre- and post-operative anorectal manometry, CAES leads to a statistically significant increase in the maximum defecation pressure, a significant decrease in the rectal residual pressure, and a significant increase in the relaxation rate. There were no severe adverse side effects reported in this study. Another interesting aspect of rectal prolapse is the management of irreducible rectal prolapse that presents as a surgical emergency. In such a case, the rectum becomes edematous, it begins to ulcerate, and it cannot be reduced manually by the patient [31]. Seenivasagam et al. [31] reported a case series of 15 patients who presented with irreducible rectal prolapse from 2006 to 2010. In five of these patients, reduction was achieved by gentle manipulation under analgesia, while in two cases, reduction was achieved after applying sugar to the prolapsed rectum. The remaining cases were treated under general anesthesia with various techniques applied. In one case, general anesthesia was enough for successful reduction. One more patient underwent Delorme’s procedure, and two more underwent laparotomy and Well’s repair. The remaining cases were managed by either abdominal or perineal bowel resection. 

In the latest guidelines published by the American Society of Colorectal Surgeons, it is mentioned that sigmoidectomy can be performed in patients who suffer from rectal prolapse and constipation, in addition to posterior suture rectopexy (recommendation: 1B) [1]. Furthermore, it is noted that improvement of fecal incontinence rate may be lower when sigmoidectomy is carried out [1]. Nonetheless, our results suggest that sigmoid resection does not affect the postoperative functional outcomes. While it is widely accepted that rectopexy is essential for the operative management of full-thickness rectal prolapse through the peritoneal approach, in the same guidelines, it is mentioned that there is no evidence that the use of different kinds of meshes for the rectopexy is superior to the sutures alone [1]. In our meta-analysis, mesh placement was found not to be superior to resection rectopexy. As a consequence, and taking into account the lack of consensus on the optimal surgical technique for the management of rectal prolapse, an individualized approach for each patient is required, while the surgeon’s preference and expertise will also play a major role. Clinicians should take into account each patient’s specific symptoms, anatomy, and bowel habits, as well as their pre-operative expectations. A thorough pre-operative workup will also be a deciding factor and should include physical examination, colonoscopy and proctoscopy, and, in specific cases, defecography and anal manometry.

This study has certain limitations. The articles included in the analysis were not randomized clinical trials, and there was no blinding of the researchers involved. This lack of randomization may have introduced a selection bias as, in such cases, patients tend to be allocated to the treatment that seems more beneficial to them. Other forms of bias that could have potentially affected our outcomes include bias due to missing data as a result of patients being lost to follow-up and bias in the selection of the reported results that prevents the estimate from being included in the meta-analysis. Moreover, lack of blinding could have introduced performance bias and bias in the measurement of the outcomes with an overestimation of the treatment effect as the assessors were aware of the intervention status. Also, despite including a total number of eight studies, not all of them provided extractable data to be included in every comparison performed. 

## 5. Conclusions

The findings of our article show that mesh rectopexy and resection rectopexy for rectal prolapse have comparable outcomes, with neither of these methods demonstrating any superiority over the other in terms of complication rate, long-term outcomes, and recurrence rate. Therefore, the operative approach selected should mainly rely on the surgeon’s preference and expertise. However, as indicated by the trial sequential analysis performed, more studies are required to consolidate our findings. We suggest that future researchers focus on performing randomized trials with independent assessors evaluating the outcomes of the procedures. We also suggest that these assessors are blinded to the original surgical technique that was utilized for each case.

## Figures and Tables

**Table 1:** Primary characteristics of the selected articles [12,13,14,15,16,17,18,19].

Authors	Study Type	Resection Rectopexy (*n*)	Mesh Rectopexy (*n*)	Age in the Resection Group [Mean ± SD or Median (min, max)]	Age in the Mesh Group [Mean ± SD or Median (min, max)]	Sex (Male/Female)
McKee et al. [16]	Prospective, randomized	9	9	69 ± 4	70 ± 4	4/14
Luukkonen et al. [15]	Prospective, randomized	15	15	65.6	66.8	2/28
Benoist et al. [13]	Retrospective	18	14	53.5 ± 20.8	66.3 ± 17.3	3/29
Demirbas et al. [14]	Retrospective	13	20	25.3 (21–33)	24.7 (19–57)	31/2
Lechaux et al. [17]	Retrospective	13	35	53 (18–87)		4/44
Senapati et al. [19]	Prospective, randomized	32	35	58 ± 18	58 ± 16	10/68 (10 patients lost in follow-up)
Forminje Jonkers et al. [18]	Retrospective	28	40	50.1 ± 17.9	67.0 ± 15.4	4/64
Carvalho et al. [12]	Retrospective	79	108	53.86 ± 19.33	59.03 ± 17.0	12/175

## Data Availability

The data supporting the findings of this study are available within the article.
